# Systematic Design of a Metal Ion Biosensor: A Multi-Objective Optimization Approach

**DOI:** 10.1371/journal.pone.0165911

**Published:** 2016-11-10

**Authors:** Chih-Yuan Hsu, Bor-Sen Chen

**Affiliations:** Lab of Control and Systems Biology, Department of Electrical Engineering, National Tsing Hua University, Hsinchu, 30013, Taiwan; Consiglio Nazionale delle Ricerche, ITALY

## Abstract

With the recent industrial expansion, heavy metals and other pollutants have increasingly contaminated our living surroundings. Heavy metals, being non-degradable, tend to accumulate in the food chain, resulting in potentially damaging toxicity to organisms. Thus, techniques to detect metal ions have gradually begun to receive attention. Recent progress in research on synthetic biology offers an alternative means for metal ion detection via the help of promoter elements derived from microorganisms. To make the design easier, it is necessary to develop a systemic design method for evaluating and selecting adequate components to achieve a desired detection performance. A multi-objective (MO) H_2_/H_∞_ performance criterion is derived here for design specifications of a metal ion biosensor to achieve the H_2_ optimal matching of a desired input/output (I/O) response and simultaneous H_∞_ optimal filtering of intrinsic parameter fluctuations and external cellular noise. According to the two design specifications, a Takagi-Sugeno (T-S) fuzzy model is employed to interpolate several local linear stochastic systems to approximate the nonlinear stochastic metal ion biosensor system so that the multi-objective H_2_/H_∞_ design of the metal ion biosensor can be solved by an associated linear matrix inequality (LMI)-constrained multi-objective (MO) design problem. The analysis and design of a metal ion biosensor with optimal I/O response matching and optimal noise filtering ability then can be achieved by solving the multi-objective problem under a set of LMIs. Moreover, a multi-objective evolutionary algorithm (MOEA)-based library search method is employed to find adequate components from corresponding libraries to solve LMI-constrained MO H_2_/H_∞_ design problems. It is a useful tool for the design of metal ion biosensors, particularly regarding the tradeoffs between the design factors under consideration.

## Introduction

Metal ion pollutants are commonly found in soil, water, and crops. With the recent industrial expansion, wastewater containing heavy metal increasingly contaminates our living surroundings [1–4]. Furthermore, non-degradable heavy metals may accumulate in food chains, and the resulting toxicity damages organisms [5–7]. Hence, detection techniques have gradually begun to receive attention. Recent progress in research on synthetic biology offers an alternative means for metal ion detection via the help of promoter elements, such as P*cusC* and P*pcoE* derived from *E*. *coli* [8] or P*pbrA* acquired from *R*.*metallidurans* [9, 10]. To make the design of detectors easier, it is necessary to develop a method to evaluate and select adequate components for achieving a desired detection performance.

In recent years, large numbers of genetic tools and engineering approaches have been and still are being developed for metal ion biosensors. Synthetic biologists are forced to find interchangeable parts, such as promoters, ribosome binding sites (RBSs), and regulatory sequences, that can be validated as construction units and assemble devices. The ability to quickly and reliably engineer biological systems from libraries of standard interchangeable parts is one trademark of modern technology [11–15]. Thus, to build a metal ion biosensor for a specified purpose, one may need a systematic design process that begins with the specification, which states the desired goal and technical details. Based on the specification, the biosensor is then represented by a block diagram which consists of functional units of the system. At later stage the design is evaluated and verified its feasibility via computational simulations and experimental validations until the configuration and combination of biological parts reach suitable performance [16]. Although a great deal has been accomplished in a short time, engineering a metal ion biosensor to produce a desired behavior still remains an acute problem, due to the uncertainties and fluctuations at the molecular level [17–29].

Recently, applying the analysis of nonlinear stochastic molecular systems to evaluate the flexibility of combinations of biological parts has been a subject of considerable interest. A multi-objective H_2_/H_∞_ performance criterion is derived here for the design specifications of a metal ion biosensor to achieve the H_2_ optimal tracking of a desired I/O response and H_∞_ optimal attenuation of parameter fluctuations and cellular noise simultaneously. Based on the design specifications, the optimal design of the biosensor can be solved by an associated Hamilton Jacobi inequality (HJI)-constrained optimization problem, which cannot be easily achieved by present analytical or numerical methods. In order to simplify the analysis and design of a nonlinear stochastic metal ion biosensor with multi-objective H_2_/H_∞_ performance, a Takagi-Sugeno (T-S) fuzzy model is employed here to interpolate several local linear stochastic systems to approximate the nonlinear stochastic metal ion biosensor system. This allows the HJI-based design problem to be replaced by a linear matrix inequality (LMI)-based design problem. Thus, the multi-objective H_2_/H_∞_ I/O response matching design of a synthetic biosensor then can be achieved by solving a LMIs-constrained multi-objective optimization problem.

However, there are tradeoffs between the H_2_ and H_∞_ performances. As natural selection is an important mechanism in defining traits best suited to environmental change in the face of evolutionary trade-offs [30], one question that arises is whether a similar strategy could be adopted for multi-objective design problems. Inspired by biological evolution events, such as mutation, crossover, and selection, a multi-objective evolutionary algorithm (MOEA) is a method to determine non-dominated Pareto optimal solutions [31, 32]. In particular, MOEA is useful when considering the tradeoffs between design factors under consideration in multi-objective H_2_/H_∞_ design problems. Consequently, according to the criterion required for the user-oriented specifications, the design can be constructed by selecting adequate components with the help of a multi-objective evolutionary algorithm (MOEA)-based searching method. In summary, this study provides a systematic design method for developing next-generation synthetic biology, from biological component selection to genetic circuit assembly. When the component libraries are more complete, more precise detection for metal ion can be achieved.

The contributions of this paper are fourfold. (a) A nonlinear stochastic system is introduced to model a metal ion biosensor with intrinsic parameter fluctuations and extrinsic molecule noise. (b) A multi-objective (MO) H_2_/H_∞_ I/O response matching performance criterion is derived to fit the design specification for a metal ion biosensor, which achieves the H_2_ optimal tracking of a desired I/O response and H_∞_ optimal robust attenuation of parameter fluctuations and cellular noise simultaneously. (c) By solving a LMIs-constrained optimization problem, a metal ion biosensor can be constructed, which achieves the H_2_/H_∞_ multi-objective design by selecting adequate components from existing libraries. (d) The proposed MOEA-based search method provides synthetic biologists with a useful tool for the design of metal ion biosensors, particularly in the face of tradeoffs between the design factors considered in next-generation synthetic biology.

## Materials and Methods

**Fig 1:**
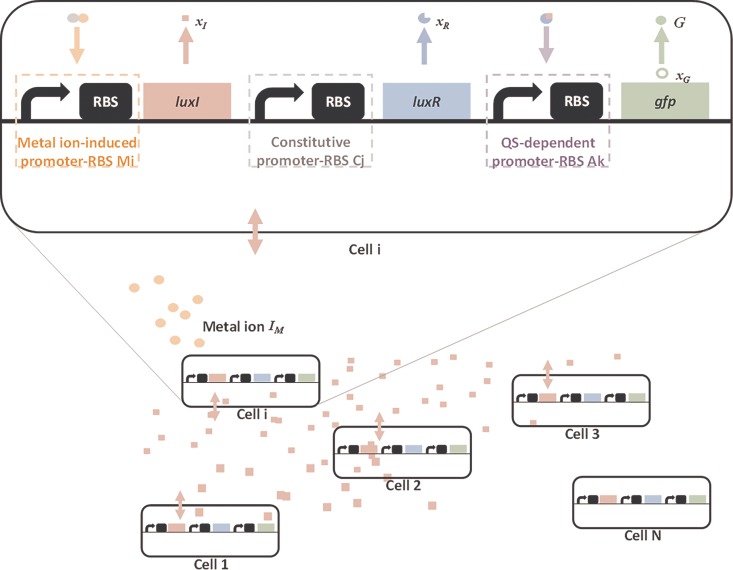
A metal ion biosensor. The metal ion biosensor is assembled by selecting a set of promoter-RBS components from the corresponding component libraries in S1 File, namely, a metal ion-induced promoter-RBS component *M*_*i*_ from the component library in Table A in S1 File, a constitutive promoter-RBS component *C*_*j*_ from the component library in Table B in S1 File, and a QS-dependent promoter-RBS component *A*_*k*_ from the component library in Table C in S1 File.

For the convenience of description and explanation, as shown in Fig 1, the metal ion biosensor is assembled by selecting a set of promoter-RBS components from the corresponding component libraries in S1 File. The assembly included a metal ion-induced promoter-RBS component *M*_*i*_ from the component library in Table A in S1 File, a constitutive promoter-RBS component *C*_*j*_ from the component library in Table B in S1 File, and a quorum sensing (QS)-dependent promoter-RBS component *A*_*k*_ from the component library in Table C in S1 File. The metal ion-induced promoter-RBS *M*_*i*_ connects downstream with the LuxI coding sequence, the production of which synthesizes a specific N-acylated homoserine lactone (AHL) as a signal molecule [33–35]. The LuxR coding sequence is connected to the constitutive promoter-RBS component *C*_*i*_. When a sufficient amount of the LuxR protein is produced in the presence of AHL, AHL binds to the LuxR protein to form a complex [34, 36, 37]. The complex targets the cognate QS-dependent promoter-RBS component *A*_*k*_ and thereby activates transcription of the green fluorescent protein (GFP) coding sequence.

The dynamic model of the metal ion biosensor in Fig 1 can then be described as follows:

$$\left\{ \begin{array}{l} {\dot{x}}_{E}(t)=P_{M}(P_{u,i},P_{l,i},x_{S},I_{M})-(d+r_{E})\cdot x_{E}(t)\\ {\dot{x}}_{I}(t)=ax_{E}(t)-(d+r_{I})\cdot x_{I}(t)\\ {\dot{x}}_{R}(t)=P_{C}(P_{u,j},0,0,0)-(d+r_{R})\cdot x_{R}(t)\\ {\dot{x}}_{G}(t)=P_{A}(P_{u,k},P_{l,k},x_{R},x_{I})-(d+r_{G})\cdot x_{G}(t)\\ \dot{G}(t)=mx_{G}-(d+r_{O})\cdot G(t) \end{array}\right.$$ (1)

in which

$$\begin{array}{l} P_{C}(P_{u,j},0,0,0)=P_{u,j}\\ P_{M}(P_{u,i},P_{l,i},x_{S},I_{M})=P_{u,i}+\frac{P_{u,i}-P_{l,i}}{1+{\left(\frac{K_{SI}}{x_{SI}(x_{S},I_{M})}\right)}^{n_{SI}}}\\ P_{A}(P_{u,k},P_{l,k},x_{R},x_{I})=P_{u,k}+\frac{P_{u,k}-P_{l,k}}{1+{\left(\frac{K_{RI}}{x_{RI}(x_{R},x_{I})}\right)}^{n_{RI}}}\\ x_{SI}(x_{S},I_{M})=\frac{x_{S}}{1+\left(\frac{K_{M}}{I_{M}}\right)},x_{RI}(x_{R},x_{I})=\frac{x_{R}}{1+\left(\frac{K_{I}}{x_{I}}\right)} \end{array}$$

where *x*_*E*_, *x*_*I*_, *x*_*R*_, and *x*_*G*_ denote the concentrations of autoinducer synthase, autoinducer, transcriptional activator protein, and immature reporter protein, respectively, and *G* denotes the intensity of GFP fluorescence. *I*_*M*_ is the concentration of metal ions and *x*_*S*_ is the total concentration of the metal ion-dependent regulatory protein. *x*_*SI*_ denotes the complex of *x*_*I*_ and *I*_*M*_, while *x*_*RI*_ represents the complex of *x*_*R*_ and *x*_*I*_. *P*_*M*_(*P*_*u*,*i*_,*P*_*l*,*i*_ *x*_*S*_,*I*_*M*_), *P*_*C*_(*P*_*u*,*i*_,0,0,0), and *P*_*A*_(*P*_*u*,*k*_,*P*_*l*,*k*_,*x*_*R*_,*x*_*I*_) are the activities of the metal ion-induced promoter-RBS component, the constitutive promoter-RBS component, and the QS-dependent promoter-RBS component, respectively. *P*_*u*,*i*_ and *P*_*l*,*i*_ are the maximum and minimum promoter-RBS strengths of the *i*^*th*^ metal ion-induced promoter-RBS component in Table A in S1 File; *P*_*u*,*j*_ is the promoter-RBS strength of the *j*^*th*^ constitutive promoter-RBS component in Table B in S1 File, and *P*_*u*,*k*_ and *P*_*l*,*k*_ are the maximum and minimum promoter-RBS strengths of the *k*^*th*^ QS-dependent promoter-RBS component in Table C in S1 File. *r*_*E*_ denotes the degradation rate for autoinducer synthase, *r*_*I*_ denotes the degradation rate for the autoinducer itself, *r*_*R*_ denotes the degradation rate for the transcriptional activator protein, *r*_*G*_ denotes the degradation rate for the immature reporter protein, and *r*_*O*_ denotes the degradation rate for the mature reporter protein. *d* is the dilution rate due to cell growth. *a* is the autoinducer synthesis rate. *m* is the maturation rate for the reporter protein. *K*_*SI*_ and *n*_*SI*_ denote the binding affinity and binding cooperativity between the *x*_*SI*_ complex and the corresponding promoter-RBS component, respectively. *K*_*M*_ is the dissociation rate between the metal ion *I*_*M*_ and the metal regulatory protein *x*_*S*_. *K*_*RI*_ and *n*_*RI*_ are the binding affinity and binding cooperativity between the *x*_*RI*_ complex and the promoter-RBS part, respectively. *K*_*I*_ is the dissociation rate between the autoinducer *x*_*I*_ and the transcriptional activator protein *x*_*R*_.

However, biological components are inherently uncertain in a molecular biological system. For example, the kinetic parameters of the components, including the processes of transcription and translation, the degradation rates of regulatory proteins, dilution rates of the cells, and the maturation rates for the reporter proteins, are all stochastically uncertain *in vivo* as a result of gene expression noise from biochemical processes, thermal fluctuations, DNA mutation, and evolution. Additionally, a synthetic gene circuit *in vivo* also suffers from environmental molecular noise. Therefore, the equations in (1) should be modified as follows:

$$\begin{array}{l} \left(\begin{array}{c} {\dot{x}}_{E}(t)\\ {\dot{x}}_{I}(t)\\ {\dot{x}}_{R}(t)\\ {\dot{x}}_{G}(t)\\ \dot{G}(t) \end{array}\right)=\left(\begin{array}{c} P_{M}(P_{u,i},P_{l,i},x_{S},I_{M})-(d+r_{E})\cdot x_{E}(t)\\ ax_{E}(t)-(d+r_{I})\cdot x_{I}(t)\\ P_{C}(P_{u,j},0,0,0)-(d+r_{R})\cdot x_{R}(t)\\ P_{A}(P_{u,k},P_{l,k},x_{R},x_{I})-(d+r_{G})\cdot x_{G}(t)\\ mx_{G}-(d+r_{O})\cdot G(t) \end{array}\right)\\ \;+\left(\begin{array}{c} P_{M}(\Delta P_{u,i},\Delta P_{l,i},x_{S},I_{M})-(\Delta d+\Delta r_{E})\cdot x_{E}(t)\\ \Delta ax_{E}(t)-(\Delta d+\Delta r_{I})\cdot x_{I}(t)\\ P_{C}(\Delta P_{u,j},0,0,0)-(\Delta d+\Delta r_{R})\cdot x_{R}(t)\\ P_{A}(\Delta P_{u,k},\Delta P_{l,k},x_{R},x_{I})-(\Delta d+\Delta r_{G})\cdot x_{G}(t)\\ \Delta mx_{G}-(\Delta d+\Delta r_{O})\cdot G(t) \end{array}\right)n(t)+\left(\begin{array}{c} v_{1}(t)\\ v_{2}(t)\\ v_{3}(t)\\ v_{4}(t)\\ v_{5}(t) \end{array}\right) \end{array}$$ (2)

where Δ*P*_*u*,*i*_, Δ*P*_*l*,*i*_, Δ*P*_*u*,*j*_, Δ*P*_*u*,*k*_, Δ*P*_*l*,*k*_, Δ*r*_*E*_, Δ*r*_*I*_, Δ*r*_*R*_, Δ*r*_*G*_, Δ*r*_*O*_, Δ*a*, Δ*m*, and Δ*d* are the standard deviations of the corresponding stochastic parameters and *n*(*t*) is Gaussian noise, which has a mean of zero and unit variance, and accounts for sources of random fluctuation. The Gaussian noise parameters *v*_*p*_, *p* = 1, 2, 3, 4, with a zero mean and variance of *σ*_*p*_^2^, are molecular noise for both the transcriptional and translational gene expression processes. *v*_5_ denotes molecular noise in mature protein expression.

Consequently, the whole QS-based metal ion biosensor is expressed by (2), which can also be represented by the more generalized nonlinear ordinary differential equation:

$$\begin{array}{l} \dot{x}(t)=f(x(t),S,I_{M})+f_{w}(x(t),S,I_{M})n(t)+Hv(t)\\ y(t,S)=Cx(t) \end{array}$$ (3)

where *x*(*t*) represents the state vector of the QS-based metal ion biosensor. *y*(*t*,*S*) is the output vector. *v*(*t*) is extrinsic molecular noise from the environment. *S =* (*M*_*i*_,*C*_*j*_,*A*_*k*_) is the set of promoter-RBS components selected from the corresponding component libraries in Tables A, B, and C in S1 File. *f*(*x*(*t*),*S*,*I*_*M*_) is a smooth nonlinear function that characterizes the behavior of the QS-based metal ion biosensor. *f*_*w*_(*x*(*t*),*S*,*I*_*M*_)*n*(*t*) is the intrinsic parameter fluctuations of the QS-based metal ion biosensor. *H* denotes the noise-coupling matrix. *C* is the output matrix. For the convenience of analysis and design of the QS-based metal ion biosensor inserted into host cells, the nonlinear stochastic differential equation of metal ion biosensor in (3) can be represented by the following Ito’s stochastic differential equation:

$$\begin{array}{l} dx(t)=\left(f(x(t),S,I_{M})+Hv(t)\right)dt+f_{w}(x(t),S,I_{M})dw(t)\\ y(t,S)=Cx(t) \end{array}$$ (4)

where *w*(*t*) is a standard Wiener process or Brownian motion with *dw*(*t*) = *n*(*t*)*dt* to represent the random parameter fluctuations of the synthetic gene circuit. In general, *x*(*t*) in (4) is dependent on *I*_*M*_, i.e., the solution of (4) can be represented by *x*(*t*, *I*_*M*_). If the output *y*(*t*,*S*) is the last state of metal ion biosensor, then *C* = [0,0,0,0,1].

The purpose of our design is to construct a metal ion biosensor by selecting a set of suitable components from the corresponding libraries to achieve optimal matching of a desired I/O response and minimize the effect of external disturbance and noise simultaneously within a feasible range of metal ion concentrations, i.e., to achieve optimal H_2_ matching and optimal H_∞_ disturbance filtering simultaneously. To achieve this, the following design specifications are needed:

- A reference model with the desired I/O response to be matched by the metal ion biosensor in (4) is given as follows:

$$\begin{array}{l} dx_{r}(t)=\left(A_{r}x_{r}(t)+r(t,I_{M})\right)dt\\ y_{r}(t)=C_{r}x_{r}(t) \end{array}$$ (5)

where *x*_*r*_(*t*) is the desired reference state, *y*_*r*_(*t*) is the output vector of the desired reference model, *r*(*t*,*I*_*M*_) represents a desired steady state trajectory for *x*(*t*), *A*_*r*_ is a matrix to be specified for the transient behavior of *x*_*r*_(*t*), and *C*_*r*_ is the output matrix of the desired reference model. In general, *C* = *C*_*r*_. At the steady state, *x*_*r*_(*t*) = -*A*_*r*_^-1^*r*(*t*,*I*_*M*_). If we set *A*_*r*_ = -*I*, then at the steady state, *x*_*r*_(*t*,*I*_*M*_) = *r*(*t*,*I*_*M*_). Therefore, if the desired steady state *x*_*r*_(*t*,*I*_*M*_) of the metal ion biosensor in (4) is set as *r*(*t*,*I*_*M*_) and we could select an adequate set *S* of components from the corresponding libraries so that the stochastic dynamic Eq (4) of the metal ion biosensor could match the desired reference model in (5), i.e., at the steady state, *x*_*r*_(*t*) = -*A*_*r*_^-1^*r*(*t*,*I*_*M*_) and the I/O response is given by *y*_*r*_ = -*C*_*r*_*A*_*r*_^-1^*r*(*t*,*I*_*M*_).

- Standard derivations of molecular noise and parameter fluctuations in (2) to be tolerated *in vivo* are specified in order to guarantee the robust design of the metal ion biosensor.
- H_2_ design performance between the engineered biosensor output *y* in (4) and the desired reference output *y*_*r*_ is given by:

$$\begin{array}{l} J_{2}(S)=E\int {\left(y_{r}(t,S)-y(t)\right)}^{T}Q\left(y_{r}(t,S)-y(t)\right)dt\\ \;=E\int \bar{y}{\left(t,S\right)}^{T}\bar{Q}\bar{y}(t,S)dt \end{array}$$ (6)

where *Q* is the weighting matrix and $\bar{y}$ is the output of the following augmented system:

$$\begin{array}{l} d\bar{x}(t)=\left(\bar{f}(\bar{x}(t),S,I_{M})+\bar{H}\bar{v}(t)\right)dt+{\bar{f}}_{w}(\bar{x}(t),S,I_{M})d\bar{w}(t)\\ \bar{y}(t,S)=\bar{C}\bar{x}(t) \end{array}$$ (7)

and

$$\begin{array}{l} \bar{x}(t)=\left(\begin{array}{c} x(t)\\ x_{r}(t) \end{array}\right),\;\bar{y}(t)=\left(\begin{array}{c} y(t)\\ y_{r}(t) \end{array}\right),\;\bar{v}(t)=\left(\begin{array}{c} v(t)\\ r(t) \end{array}\right),\;\bar{C}=\left(\begin{array}{cc} C & 0\\ 0 & C_{r} \end{array}\right),\;\bar{H}=\left(\begin{array}{cc} H & 0\\ 0 & I \end{array}\right),\\ \bar{f}(t,S,I_{M})=\left(\begin{array}{c} f(t,S,I_{M})\\ A_{r} \end{array}\right),\;{\bar{f}}_{w}(t,S,I_{M})=\left(\begin{array}{c} f_{w}(t,S,I_{M})\\ 0 \end{array}\right),\;\bar{Q}=\left(\begin{array}{ll} -Q & \;Q\\ \;Q & -Q \end{array}\right) \end{array}$$

Since the reference signal *r*(*t*) is treated as an uncertain external input by the designer, it account for sources of noise.

- H_∞_ filtering performance to attenuate the effect of $\bar{v}$(*t*) on matching error is given as follows:

$$\begin{array}{l} J_{\infty }(S)=\frac{E\int {\left(y_{r}(t,S)-y(t)\right)}^{T}Q\left(y_{r}(t,S)-y(t)\right)dt}{E\int \bar{v}{\left(t\right)}^{T}\bar{v}(t)dt}\\ \;=\frac{E\int \bar{y}{\left(t,S\right)}^{T}\bar{Q}\bar{y}(t,S)dt}{E\int \bar{v}{\left(t\right)}^{T}\bar{v}(t)dt} \end{array}$$ (8)

Thus, if the H_2_ matching performance and H_∞_ filtering performance in (6) and (8) are minimized simultaneously by choosing an appropriate set of components from the corresponding component libraries in S1 File, the engineered metal ion biosensor will then optimally match the specified I/O response and optimally filter parameter fluctuations and environmental disturbances simultaneously, i.e., to select a component set *S* from component libraries in S1 File to solve the following simultaneous minimization problem:

$$\underset{S}{\min}\left(J_{2}(S),J_{\infty }(S)\right)$$ (9)

where *J*_2_(*S*) and *J*_∞_(*S*) are defined in (6) and (8), respectively. To make the design easier, an indirect method is proposed by simultaneously minimizing the upper bounds of *J*_2_(*S*) and *J*_∞_(*S*), i.e., the multi-objective problem in (9) is transformed to a suboptimal problem as follows:

$$\left(\alpha^{*},\beta^{*}\right)=\underset{S}{\min}\left(\alpha ,\beta \right)$$ (10)

subject to

$$J_{2}(S)=E\;\int \bar{y}{\left(t,S\right)}^{T}\bar{Q}\bar{y}(t,S)dt\leq \alpha$$ (11)

$$J_{\infty }(S)=\frac{E\int \bar{y}{\left(t,S\right)}^{T}\bar{Q}\bar{y}(t,S)dt}{E\int \bar{v}{\left(t\right)}^{T}\bar{v}(t)dt}\leq \beta$$ (12)

where *α* and *β* are the upper bounds of H_2_ and H_∞_ performances, respectively.

**Remark 1:** H_2_ performance in (6) can be considered as the penalty of the quadratic matching error under the assumption $\bar{v}$(*t*) ≡ 0 and *α* in (11) denotes the upper bound of H_2_ performance under the assumption $\bar{v}$(*t*) ≡ 0.

**Remark 2:** The inequality in (12) means that the effect of extrinsic molecular noise on the matching error is less than *β* from an average energy point of view. Because the statistics of extrinsic molecular noise may be unavailable or uncertain, it is very difficult to obtain the noise filtering ability β* for all possible extrinsic noise $\bar{v}$(*t*) directly and only the upper bound *β* of the noise-filtering ability β* can be given in (12) at first. Similarly, the upper bound *α* of α^*^ is also given. We will then decrease the upper bound (*α*, *β*) to as small a value as possible to approach the lower bound (α^*^, β^*^), i.e., to get (α^*^, β^*^) by minimizing (*α*, *β*) indirectly.

**Remark 3:** If the extrinsic environmental molecular noise $\bar{v}$(*t*) is deterministic, then the expectation on $\bar{v}$(*t*) in (11) and (12) should be disregarded.

**Remark 4:** If the initial condition $\bar{x}$(0) is considered, then the noise-filtering upper bound in (12) should be modified as follows:

$$E\int \bar{y}{\left(t,S\right)}^{T}\bar{Q}\bar{y}(t,S)dt\leq EV(\bar{x}(0))+\beta E\int \bar{v}{\left(t\right)}^{T}\bar{v}(t)dt$$ (13)

for some Lyapunov function *V*($\bar{x}$(0)), i.e., the energy due to the initial condition $\bar{x}$(0) should be considered in the effect of noise [38, 39].

Based on the multi-objective H_2_/H_∞_ design criterion, we obtain the following result for the QS-based metal ion biosensor design.

**Proposition 1:** The multi-objective I/O matching problem in (10)–(12) is equivalent to how to select components *M*_*i*_, *C*_*j*_, and *A*_*k*_ of the metal ion biosensor from the corresponding component libraries in S1 File to solve the following HJI-constrained multi-objective problem:

$$\left(\alpha^{*},\beta^{*}\right)=\underset{M_{i},C_{j},A_{k}}{\min}\left(\alpha ,\beta \right)$$ (14)

subject to

$$\begin{array}{l} {\bar{x}}^{T}{\bar{C}}^{T}\bar{Q}\bar{C}\bar{x}+\frac{1}{2}{\bar{f}}_{w}{\left(\bar{x},S,I_{M}\right)}^{T}\frac{\partial^{2}V(\bar{x})}{\partial {\bar{x}}^{2}}{\bar{f}}_{w}(\bar{x},S,I_{M})\\ \;+{\left(\frac{\partial V(\bar{x})}{\partial \bar{x}}\right)}^{T}{\bar{f}}_{w}(\bar{x},S,I_{M})<0 \end{array}$$

$$\begin{array}{l} {\bar{x}}^{T}{\bar{C}}^{T}\bar{Q}\bar{C}\bar{x}+\frac{1}{2}{\bar{f}}_{w}{\left(\bar{x},S,I_{M}\right)}^{T}\frac{\partial^{2}V(\bar{x})}{\partial {\bar{x}}^{2}}{\bar{f}}_{w}(\bar{x},S,I_{M})\\ \;+{\left(\frac{\partial V(\bar{x})}{\partial \bar{x}}\right)}^{T}{\bar{f}}_{w}(\bar{x},S,I_{M})+\frac{1}{4\beta }{\left(\frac{\partial V(\bar{x})}{\partial \bar{x}}\right)}^{T}\bar{H}{\bar{H}}^{T}\left(\frac{\partial V(\bar{x})}{\partial \bar{x}}\right)<0 \end{array}$$

with *V*($\bar{x}$(*t*))>0 and *EV*($\bar{x}$(0))<*α*, i.e., the I/O response of an engineered metal ion biosensor will optimally match the specified I/O response of the reference model and optimally filter intrinsic fluctuations and external disturbances simultaneously.

**Proof:** See S2 File.

It is still very difficult to solve the constrained multi-objective minimization in (14) to simultaneously achieve the optimal matching of the specified reference output in (5) and optimal filtering of parameter fluctuations and environmental disturbances. Recently, the fuzzy dynamic model has been widely used to interpolate local dynamic models to efficiently approximate a nonlinear dynamic system [40, 41]. Hence, in this situation, we employ the T-S fuzzy model to interpolate several linear systems at different local operation points to efficiently and globally approximate the augmented nonlinear system in (7) so that the design procedure for a multi-objective optimal design of a synthetic metal ion biosensor can be simplified.

In this study, the T-S fuzzy method is employed to simplify the analysis and design procedure for the QS-based metal ion biosensor under intrinsic parameter fluctuations and extrinsic environmental molecular noise. The T-S fuzzy model is described by fuzzy if-then rules. The *p*^th^ rule of the fuzzy model for the augmented system in (7) is proposed in the following form [38, 40, 41]:

$$\begin{array}{l} \mathrm{Rule}\;p\;:\;\text{If}\;z_{1}(t)\;\text{is}\;F_{p1}\;\text{and}\;z_{2}(t)\;\text{is}\;F_{p2}\;\text{and}\ldots \;\text{and}\;z_{g}(\text{t})\;\text{is}\;F_{pg}\\ \;\text{then}\;d\bar{x}(t)=\left({\bar{A}}_{p}\bar{x}(t)+\bar{H}\bar{v}(t)\right)dt+{\bar{A}}_{wp}\bar{x}(t)d\bar{w}(t)\\ \;\bar{y}(t)=\bar{C}\bar{x}(t) \end{array}$$ (15)

for *p* = 1,2,…,*L*, where *z*_*g*_ is the element of premise variables of the *p*^th^ augmented system, i.e., z = [*z*_1_,…,*z*_g_]^*T*^, *F*_*pg*_ is the fuzzy set, $\bar{A_{p}}$ and $\bar{A_{wp}}$ are the fuzzy system matrices, *L* is the number of if-then rules, and *g* is the number of premise variables. The physical meaning of the fuzzy rule *p* is that if the premise variables *z*_1_(*t*),…,*z*_g_(*t*) are with the fuzzy sets *F*_*p*1_,…,*F*_*pg*_, then the augmented system in (7) can be represented by interpolating the linearized system in (15) via the fuzzy basis. The fuzzy dynamics in (15) are denoted as follows [41–43]:

$$\begin{array}{l} d\bar{x}(t)=\sum_{p=1}^{L}\mu_{p}(z)\left(\left({\bar{A}}_{p}\bar{x}(t)+\bar{H}\bar{v}(t)\right)dt+{\bar{A}}_{wp}\bar{x}(t)d\bar{w}(t)\right)\\ \bar{y}(t)=\bar{C}\bar{x}(t) \end{array}$$ (16)

in which

$${\bar{A}}_{p}=\left(\begin{array}{cc} A_{p} & 0\\ 0 & A_{r} \end{array}\right),\;{\bar{A}}_{wp}=\left(\begin{array}{cc} A_{wp} & 0\\ 0 & 0 \end{array}\right)$$

where $\mu_{p}(z)=\prod_{q=1}^{g}F_{pq}(z_{q})/\sum_{p=1}^{L}\prod_{q=1}^{g}F_{pq}(z_{q})$, *F*_*pq*_(*z*_*q*_) is the grade of membership of *z*_*q*_ (*t*) in *F*_*pq*_ or the possibility function of *z*_*q*_ (*t*) in *F*_*pq*_, and μ_*p*_ is the fuzzy basis function for *k* = 1,2,…,*L*. The denominator $\sum_{p=1}^{L}\prod_{q=1}^{g}F_{pq}(z_{q})$ in the above fuzzy basis function is only for normalization so that the total sum of the fuzzy basis is $\sum_{p=1}^{L}\mu_{p}(z)=1$. The physical meaning of (16) is that the fuzzy stochastic system interpolates L local linear stochastic systems through the nonlinear basis μ_*p*_(*z*) to approximate the nonlinear stochastic system in (7).

**Remark 5:** In [40], Takagi and Sugeno proposed a systematic method to build a T-S fuzzy model for nonlinear function approximation by a system identification tool, i.e., the local system matrices *A*_*p*_ and *A*_*wp*_ in (16) can be identified by the least square estimation method. Conversely, many studies have proved that the T-S fuzzy model can approximate a continuous function to any degree of accuracy. However, there is still some fuzzy approximation error in (16). In the design, for simplicity, the fuzzy approximation error can be merged into the external noise.

After investigating the approximation of the nonlinear stochastic QS-based metal ion biosensor by the fuzzy interpolation method, in order to avoid solving the nonlinear constrained simultaneous optimization problem in (14) for the multi-objective design problem of a QS-based metal ion biosensor under intrinsic parameter fluctuations and extrinsic molecular noise, the measurement procedure for the matching and filtering abilities of a QS-based metal ion biosensor could also be simplified by the fuzzy approximation method. We then get the following result.

**Proposition 2:** Based on the T-S fuzzy model in (16), the H_2_/H_∞_ I/O response matching problem in Proposition 1 becomes how to select promoter-RBS components *M*_*i*_, *C*_*j*_, and *A*_*k*_ from the corresponding component libraries in S1 File to solve the following multi-objective problem:

$$\left(\alpha^{*},\beta^{*}\right)=\underset{M_{i},C_{j},A_{k}}{\min}\left(\alpha ,\beta \right)$$ (17)

subject to

$$\bar{x}{\left(0\right)}^{T}P\bar{x}(0)-\alpha \leq 0$$ (18)

$${\bar{C}}^{T}\bar{Q}\bar{C}+P{\bar{A}}_{p}+{\bar{A}}_{p}{}^{T}P+{\bar{A}}_{wp}{}^{T}P{\bar{A}}_{wp}<0$$ (19)

$$\left(\begin{array}{cc} {\bar{C}}^{T}\bar{Q}\bar{C}+P{\bar{A}}_{p}+{\bar{A}}_{p}{}^{T}P+{\bar{A}}_{wp}{}^{T}P{\bar{A}}_{wp} & P\bar{H}\\ {\bar{H}}^{T}P & \text{-}\beta \end{array}\right)<0$$ (20)

for *p* = 1,2,…,*L*. Based on the optimal selection of these promoter-RBS components, the I/O response of an engineered metal ion biosensor will achieve the optimal matching for the I/O response of the specified reference model and the optimal filtering of parameter fluctuations and environmental disturbances simultaneously.

**Proof:** See S3 File.

Thus, the multi-objective H_2_/H_∞_ optimal I/O response design of the QS-based metal ion biosensor obtained by solving the HJI-constrained multi-objective optimization problem in (14) could be replaced by solving the following LMI-constrained multi-objective optimizations:

$$\begin{array}{l} \;\left(\alpha^{*},\beta^{*}\right)=\underset{(M_{i},C_{j},A_{k})\in \Omega }{\min}\left(\alpha ,\beta \right)\\ \text{subject to}\;P>0\;\text{and LMIs in}\;(18)\text{-}(20) \end{array}$$ (21)

where Ω is the feasible set of promoter-RBS libraries in S1 File.

**Remark 6:** In this study, the fuzzy approximation method in (15) or (16) is only employed to simplify the analysis and design procedure via solving *P*>0 for LMIs in (21) instead of solving *V*($\bar{x}$(*t*))>0 for HJIs directly. Further, based on the fuzzy interpolation of local linear systems, i.e., replacing $\bar{f}$($\bar{x}$(*t*),*S*,*I*_*M*_) and $\bar{f_{w}}$($\bar{x}$(*t*),*S*,*I*_*M*_) by the fuzzy approximations in (16), *V*($\bar{x}$) = $\bar{x}$^*T*^*P*$\bar{x}$ is employed in in Proposition 2 to solve the HJI in Proposition 1. The HJI in Proposition 1 is replaced with a set of LMIs in Proposition 2 and we only need to solve *P*>0 for LMIs to guarantee the output of an engineered QS-based metal ion biosensor that will optimally match the specified reference I/O response in (5) and optimally filter parameter fluctuations and cellular disturbances simultaneously.

**Remark 7:** In general, it is very difficult to directly solve the LMI-constrained multi-objective optimization in (21) for a synthetic gene circuit. In this study, a MOEA-based library searching method is proposed to solve the LMI-based multi-objective I/O response-matching problem in (21) for a metal ion biosensor in sequel. In general, no unique solution exists such that *α* and *β* in (21) are minimized simultaneously. Therefore, more effort is needed for the multi-objective optimization problem in (21) to seek a set of Pareto optimal solutions, from which the designer can select the preferred option.

However, a problem remains with the tradeoff between H_2_ and H_∞_ performance. In light of evolutionary trade-offs, the mechanism of natural selection produces traits best-suited for adapting to environmental change. A similar strategy can be adapted for the multi-objective design problem in (21). Inspired by biological evolution, a MOEA is a population-based method to determine Pareto optimal solutions that are non-dominated. Compared with the weighted sum method, MOEA is useful for considering multi-objective design problems, in particular for assessing tradeoffs between design factors. Thus, before discussing the design procedure of the multi-objective I/O response-matching problem in (21), some properties regarding the Pareto optimal solutions are given as follows:

**Definition 1:** (Dominance) Consider two solutions (*M*_*i*_^1^, *C*_*j*_^1^, *A*_*k*_^1^) and (*M*_*i*_^2^, *C*_*j*_^2^, *A*_*k*_^2^) in Ω for two objective values (*α*_1_, *β*_1_) and (*α*_2_, *β*_2_) subject to the LMIs in (18)–(20), respectively. (*α*_1_, *β*_1_) is said to dominate (*α*_2_, *β*_2_), if *α*_1_≤*β*_1_ and *α*_2_≤*β*_2_.

**Definition 2:** (Pareto optimal solution) A solution (*M*_i_*, *C*_j_*, *A*_k_*) is the Pareto optimal solution of the multi-objective optimization problem in (21) with respect to Ω if another feasible solution does not exist (*M*_*i*_°, *C*_*j*_°, *A*_*k*_°) such that objective values (*α*°, *β*°) dominate (α*, β*).

**Definition 3:** (Pareto front) The Pareto front for the optimization problem in (21) is defined as Γ≜{(α*, β*)|(*M*_i_*, *C*_j_*, *A*_k_*). This is the Pareto optimal solution of the optimization problem in (21) and (α*, β*) is generated by (*M*_i_*, *C*_j_*, *A*_k_*) subject to the LMIs in (18)–(20)}.

The design procedure for a QS-based metal ion biosensor is then summarized as follows:

1. Provide user-defined design specifications as a desired reference model in (5) for the quorum sensing-based metal ion biosensor.
2. Select an initial set *S* of promoter-RBS components from corresponding libraries, each of which can be satisfied with the LMIs in (18)–(20) with *P*>0.
3. Sort the current set *S* into different fronts by Pareto dominance ranking and assign a crowding distance to each of them.
4. Create an offspring set *S* using MOEA operators, such as reproduction, crossover, and mutation.
5. Calculate the objective values of the new set *S* obtained by natural selection. Stop when the Pareto front is achieved or an acceptable solution is obtained. Otherwise, create the next generation and return to step 3.

**Remark 8:** In addition to the design of a QS-based metal ion biosensor, the proposed method can be applied to the design of synthetic gene regulatory networks with any kind of dynamic behavior.

## Results

The design procedure begins by representing the nonlinear stochastic augmented system of a metal ion biosensor and the desired reference model in (7) by the Takagi-Sugeno (T-S) fuzzy model in (16) using the interpolation of linear stochastic systems. In particular, at steady state, the desired fluorescence intensity of the metal ion biosensor to different metal ion concentrations is described as follows:

$$G_{ref}(I_{M})=65+\frac{5000}{1+{\left(10^{-1}/I_{M}\right)}^{2}}$$ (22)

**Fig 2:**
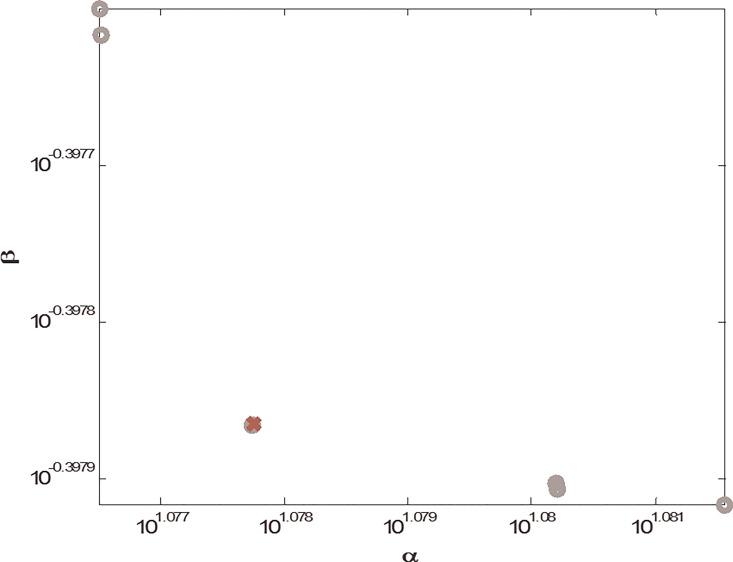
Pareto front obtained by solving the multi-objective problem in (21) through the proposed MOEA-based library search method from Tables A–C.

**Fig 3:**
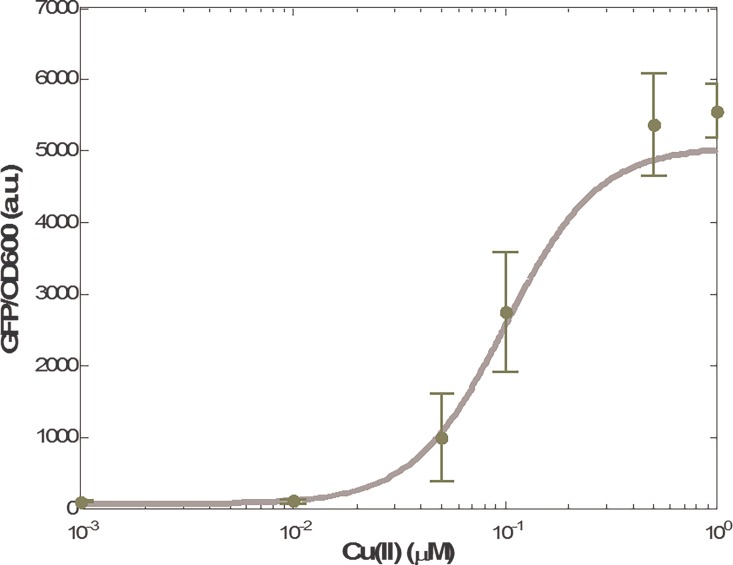
The resulting metal ion biosensor. The adequate set *S* = (*M*_1_, *C*_6_, *A*_3_) is selected from the corresponding libraries in S1 File. The green points are the experimental results (mean of three trials) by *S* = (*M*_1_, *C*_6_, *A*_3_). The gray solid line is the desired I/O response in (22).

According to (5), at steady state, our design goal for the steady state in (5) is *x*_*r*_(*t*) = –*A*_*r*_^–1^*r*(*t*,*I*_*M*_) and thereby *y*_*r*_ = –*C*_*r*_*A*_*r*_^–1^*r*(*t*,*I*_*M*_). In order to let the steady state *y*_*r*_ in (5) match *G*_*ref*_(*I*_*M*_) in (22), if we select the followings for the reference model in (5)

$$A_{r}=\text{-}I,\;C_{r}=\left(0,0,0,0,1\right),\;r(t,I_{M})={\left(0,0,0,0,G(I_{M})\right)}^{T}$$ (23)

then *y*_*r*_ = *G*_*ref*_(*I*_*M*_) at the steady state of the reference model in (5). We suppose the quorum sensing-based metal ion biosensor suffers from intrinsic parameter fluctuations, with zero mean and unit variance, as well as the external environmental noises *v*_1_, *v*_2,_ *v*_3,_ and *v*_4_ for the transcription and translation processes, and noise *v*_5_ for mature reporter protein expression, are all Gaussian, with zero mean and unit variance. In order to then efficiently achieve the desired I/O response matching design problem of the metal ion biosensor under intrinsic parameter fluctuations and external disturbances, the multi-objective H_2_/H_∞_ matching design in (9) is applied to the design problem. Based on the design procedure, a MOEA-based library search method is employed to search a set *S* from corresponding libraries in S1 File to minimize the objective values in (21) subject to *P*>0 and the LMIs in (18)–(20). From the Pareto front in Fig 2, there are six Pareto solutions. The one with the red cross that makes a compromise between the optimal H_2_ solution and H_∞_ solution is selected for the multi-objective H_2_/ H_∞_ I/O response of the metal ion biosensor. In this design case, the components from the corresponding libraries are found to be *M*_1_, *C*_3_, and *A*_3_. The desired response is shown in Fig 3, with the fluorescence intensity values under different Cu(II) ion concentrations. Clearly, at steady state, the metal ion biosensor can match the desired I/O response in (22), despite the parameter fluctuations and environmental disturbances.

## Discussion

With recent industrial expansion, heavy metal and other pollutants increasingly contaminate our living surroundings [2–4]. Heavy metals are non-degradable and may accumulate in food chains, where the resulting toxicity can damage organisms [6, 7]. Therefore, heavy metal detection techniques have gradually begun to receive attention.

In order to more easily design a QS-based metal ion biosensor, a multi-objective H_2_/H_∞_ performance criterion is derived to infer a sufficient condition required for user-oriented specifications using a direct method by minimizing the upper bound of H_2_ and H_∞_ performance simultaneously. Based on the multi-objective design criterion, a metal ion biosensor can then be designed by solving an associated HJI-constrained optimization problem. However, the HJI-constrained optimization problem is difficult to solve directly by any analytical or numerical method because of the complexity of nonlinear dynamics. Therefore, a Takagi-Sugeno (T-S) fuzzy model is employed here to solve the HJI easily and indirectly. The T-S fuzzy model has been widely applied to approximate nonlinear systems by interpolating several local linearized stochastic systems. By using a T-S fuzzy model and choosing an appropriate Lyapunov function, the HJI-constrained multi-objective optimization problem in (14) for solving the H_2_/H_∞_ I/O response matching of a nonlinear stochastic metal ion biosensor is reduced to an equivalent LMI-constrained multi-objective optimization problem in (21), which can be solved efficiently by an MOEA algorithm with the help of MATLAB’s LMI toolbox. Thus, according to the LMI-constrained criterion, the multi-objective metal ion biosensor design can be constructed by evaluating and selecting adequate promoter-RBS components from corresponding libraries within a feasible range of metal ion concentrations.

However, because the multi-objective design problem has no unique solution, a problem remains in dealing with the tradeoff between H_2_ and H_∞_ performance. In light of natural selection on traits best-suited for environmental change being an important mechanism for determining evolutionary trade-offs, a similar strategy seems to be adaptable for the multi-objective design problem. Inspired by biological evolution, the MOEA is a population-based method to determine non-dominated Pareto optimal solutions. Unlike the necessity for complicated computations in conventional design strategies, only simple operators (e.g., selection, crossover, and mutation) and some simple calculations are required for the iterative selection of adequate components. Therefore, MOEAs are useful when considering design problems, in particular for assessing tradeoffs between the design factors under consideration. Consequently, according to the user-specified criteria, this method may offer possible design guidelines for selecting adequate components for a QS-based metal ion biosensor from the corresponding libraries. When the component libraries are more complete, a more precise detection performance of metal ion biosensor can be achieved. In fact, in addition to the QS-based metal ion biosensor, the proposed method can be applied to the design of synthetic gene regulatory networks with any kind of dynamic behavior.

## Conclusion

In this study, a nonlinear stochastic system is introduced to model a synthetic metal ion biosensor with intrinsic parameter fluctuations and extrinsic molecule noise. A multi-objective H_2_/H_∞_ I/O response matching performance criterion is derived here for the design specifications of the metal ion biosensor in order to simultaneously achieve the optimal H_2_ matching of the desired I/O behavior and the optimal H_∞_ filtering of parameter fluctuations and cellular noise. An indirect method is proposed to solve the multi-objective H_2_/H_∞_ I/O response matching design by minimizing their upper bounds simultaneously. Further, based on a fuzzy interpolation technique, the HJI-constrained multi-objective design problem for the metal ion biosensor is transferred to a more simple LMI-constrained multi-objective design problem. According to the LMI-constrained multi-objective design criterion, a metal ion biosensor can be constructed with a desired I/O response by evaluating and selecting adequate components from the corresponding promoter-RBS libraries. The proposed MOEA-based search method provides synthetic biologists with a useful tool for the design of gene circuits, particularly in regards to tradeoffs between the design factors under consideration. The experimental results verify that the design can optimally match the specified reference I/O response and can optimally filter parameter fluctuations and environmental disturbances simultaneously.

## Supporting Information

S1 File Supplementary Appendix A. (DOCX)

S2 File Supplementary Appendix B. (DOCX)

S3 File Supplementary Appendix C. (DOCX)
