# Supplementary material for: Systematic Design of a Metal Ion Biosensor: A Multi-Objective Optimization Approach
Source: PLoS One. 2016 Nov 10;11(11):e0165911. doi: 10.1371/journal.pone.0165911 (PMC5104392; doi:10.1371/journal.pone.0165911)
Supplement: S1 File — (DOCX) [file pone.0165911.s001.docx]

**Supplementary Appendix A**

**Materials and methods**

All restriction enzymes and DNA ligation kit are purchased from New England Biolabs. The chemicals used here are from Sigma-Aldrich. The oligonucleotides are from Integrated DNA Technologies. Escherichia coli strain DH5α cells from Yeastern biotech (ECOS) are used for the construction of the procedure and for the fluorescence measurement experiments. An M9 working medium (34 g/L Na2HPO4, 15 g/L K2HPO4, 2.5 g/L NaCl, 5 g/L NH4Cl, 2 g/L casamino acids, 2 μM vitamin B1, 2 mM MgSO4, 0.2% glucose) with proper antibiotics is used for E. coli cultivation at 37 °C and 200 r.p.m. The DNA parts used in this study are selected from BioBrick or synthesized by MDBio Biotech Co. Ltd. All DNA parts are assembled into the backbone pSB3K3 (20–30 copies per cell), via the BioBrick standard assembly method. The cells containing the genetic circuit are inoculated in the M9 working medium with 50 mg/mL Kanamycin. The culture is incubated for approximately 14–16 hours, after which it is diluted 250-fold and further incubated until the OD600 reaches 0.1. The fluorescence intensity of the culture is measured subsequently by the microplate reader (BioTek, Synergy™ H1, GFP settings are 490/510 nm for excitation and emission).

**Construction of the promoter-RBS library**

To construct a component library, promoter-RBS regulation must be introduced first. The regulation function here is defined by *P*(*P_u_*,*P_l_*,*TF*, *I*), in which *P_u_* and *P_l_* denote the maximum and minimum promoter-RBS strengths, respectively, *TF* is transcription factor concentration, and *I* denotes inducer concentration. Thus, the expression of a reporter protein produced from a promoter-RBS component can be described by

 (A1)

where *x_G_* is the concentration of immature reporter protein; *G* is the concentration of mature reporter protein. *d* represents the dilution rate due to cell growth. *m* denotes the maturation rate for the reporter protein. *r_G_* and *r* are the degradation rates for immature reporter protein and mature reporter protein, respectively. Details of the regulation functions for metal ion-induced promoter-RBS component *M_i_*, constitutive promoter-RBS component *C_i_* and QS-dependent promoter-RBS component *A_k_* are described as follows.

1. Regulation function of the metal ion-induced promoter-RBS component

A metal-ion induced promoter-RBS component *M_i_* is regulated by regulatory protein *x_S_*, which can bind with metal ion *I_M_* and thereby influence downstream gene expression. The concentration of metal ion-bound regulatory protein can be described as follows:

$x_{SI}(x_{S},I_{M})=\frac{x_{S}}{1+\left(\frac{K_{M}}{I_{M}}\right)}$ (A2)

where *x_S_* denotes the total metal ion-dependent regulatory protein including free and metal ion-bound regulatory protein; *I_M_* denotes metal ion concentration; *K_M_* is the dissociation rate between the metal ion *I_M_* and the metal regulatory protein *x_S_*. Therefore, the regulation activity of the metal ion-induced promoter-RBS component *M_i_* can be represented by a promoter-RBS regulation function as follows:

$P_{M}(P_{u,i},P_{l,i},x_{S},I_{M})=P_{u,i}+\frac{P_{u,i}-P_{l,i}}{1+\left(\frac{K_{SI}}{x_{SI}(x_{S},I_{M})}\right)^{n_{SI}}}$ (A3)

where *i* denotes the *i^th^* metal ion-induced promoter-RBS component. *P_u,i_* and *P_l,i_* are the maximum and minimum promoter-RBS strengths of the *i^th^* metal ion-induced promoter-RBS component. *K_SI_* and *n_SI_* denote the binding affinity and binding cooperativity between the complex *x_SI_* and the corresponding promoter-RBS component, respectively. The metal ion-induced promoter-RBS components here are constructed by two copper-induced promoters, namely, P*cusC* and P*pcoE* and a RBS, namely, B0034.

1. Regulation function of the constitutive promoter-RBS component

A constitutive promoter-RBS part *C_i_* can express downstream gene constitutively, with no influence by any transcription factor. Therefore, the regulation activity of the constitutive promoter-RBS component *C_i_* can be represented by a promoter-RBS regulation function as follows:

$P_{C}(P_{u,j},0,0,0)=P_{u,j}$ (A4)

where *j* denotes the *j^th^* constitutive promoter-RBS component from the corresponding library. *P_u,j_* denotes the promoter-RBS strength of the *j^th^* constitutive promoter-RBS component. The constitutive promoter-RBS components here are constructed by three constitutive promoters, namely, J23101, J23105, and J23106, as well as three RBSs, namely, B0031, B0032 and B0034.

1. Regulation function of the QS-dependent promoter-RBS component

A QS-dependent promoter-RBS component *A_k_* is regulated by activator protein *x_R_*, which can bind with autoinducer x*_I_* to influence downstream gene expression. The concentration of inducer-bound activator can be described as follows:

$x_{RI}(x_{R},x_{I})=\frac{x_{R}}{1+\left(\frac{K_{I}}{x_{I}}\right)}$ (A5)

where *x_R_* denotes the total concentration of transcriptional activator protein; *x_I_* denotes the concentration of autoinducter. *K_I_* is the dissociation rate between the autoinducer *x_I_* and the transcriptional activator protein *x_R_*. Therefore, the regulation activity of the QS-dependent promoter-RBS component *A_k_* can be represented by a promoter-RBS regulation function as follows:

$P_{A}(P_{u,k},P_{l,k},x_{R},x_{I})=P_{u,k}+\frac{P_{u,k}-P_{l,k}}{1+\left(\frac{K_{RI}}{x_{RI}(x_{R},x_{I})}\right)^{n_{RI}}}$ (A6)

where *k* is the *k^th^* QS-dependent promoter-RBS component. *P_u,k_* and *P_l,k_* are the maximum and minimum promoter-RBS strengths of the *k^th^* QS-dependent promoter-RBS component. *K_RI_* and *n_RI_* are the binding affinity and binding cooperativity between the complex *x_RI_* and the promoter-RBS part, respectively. The QS-dependent promoter-RBS components here are constructed by a QS-dependent promoter, namely, R0062 and three RBSs, namely, B0031, B0032 and B0034.

The dynamic model is transformed into steady-state model by assuming that the dynamics in (A1) are equal to zero. The steady-state model is derived as follows:

$\left\{\begin{matrix} x_{GSS}(t)=\frac{P(P_{u},P_{l},TF,I)}{d+r_{G}} \\ G_{SS}(t)=\frac{m}{d+r}x_{G} \end{matrix}\right.$ (A7)

where *x_GSS_* and *G_SS_* are the steady-state concentrations of immature reporter protein and mature reporter protein, respectively. Then based on the steady-state model in (A7), the identification results are used to construct libraries and listed in Tables A, B and C, respectively.

Table A Metal ion-induced promoter-RBS component library in E. coli strain DH5α

| Index | Component | P_u_ | P_l_ |  |
| --- | --- | --- | --- | --- |
| M_1_ | PcusC-B0034 | 100.630 | - | K_SI_=81.167 nM  K_M_=0.3603 μM  n_SI_=1 |
| M_2_ | PpcoE-B0034 | 98.237 | - | K_SI_=154.903 nM  K_M_=0.3603 μM  n_SI_=1 |

Table B Constitutive promoter-RBS component library in E. coli strain DH5α

| Index | Component | P_u_ | P_l_ |  |
| --- | --- | --- | --- | --- |
| C_1_ | J23105-B0031 | 5.178 | - |  |
| C_2_ | J23105-B0032 | 3.449 | - |  |
| C_3_ | J23105-B0034 | 14.342 | - |  |
| C_4_ | J23106-B0031 | 8.580 | - |  |
| C_5_ | J23106-B0032 | 5.813 | - |  |
| C_6_ | J23106-B0034 | 28.874 | - |  |

Table C QS-dependent promoter-RBS component library in E. coli strain DH5α

| Index | Component | P_u_ | P_l_ |  |
| --- | --- | --- | --- | --- |
| A_1_ | R0062-B0031 | 362.902 | - | K_RI_=95.361 nM  K_I_=3.826 nM  n_RI_=1 |
| A_2_ | R0062-B0032 | 238.132 | - |  |
| A_3_ | R0062-B0034 | 848.678 | - |  |
