# Supplementary material for: Systematic Design of a Metal Ion Biosensor: A Multi-Objective Optimization Approach
Source: PLoS One. 2016 Nov 10;11(11):e0165911. doi: 10.1371/journal.pone.0165911 (PMC5104392; doi:10.1371/journal.pone.0165911)
Supplement: S2 File — (DOCX) [file pone.0165911.s002.docx]

**Supplementary Appendix B: Proof of Proposition 1**

First, in the H_2_ design case with $\overline{v}$(*t*)$\equiv0$, consider the following equality

$E\int_{0}^{t_{f}} \bar{y}^{T}\bar{Q}\bar{y}dt=EV\left(\bar{x}(0)\right)-EV\left(\bar{x}(t_{f})\right)+E\int_{0}^{t_{f}} \left(\bar{y}^{T}\bar{Q}y+\frac{dV(\bar{x})}{dt}\right)dt$ (B1)

By Ito formula, we get

$\begin{matrix} dV(\bar{x})=\left(\frac{\partial V(\bar{x})}{\partial\bar{x}}\right)^{T}\left(\bar{f}(\bar{x},S,I_{M})dt+\bar{f}_{w}(\bar{x},S,I_{M})d\bar{w}\right) \\ \text{ }+\frac{1}{2}\bar{f}_{w}(\bar{x},S,I_{M})^{T}\frac{\partial^{2}V(\bar{x})}{\partial\bar{x}^{2}}\bar{f}_{w}(\bar{x},S,I_{M})dt \end{matrix}$ (B2)

Substituting (B2) into (B1) and by the fact *Ed*$\overline{w}$=0, *EV*($\overline{x}$(*t_f_*))$\geq$0, we get

$\begin{matrix} E\int_{0}^{t_{f}} \bar{y}^{T}\bar{Q}\bar{y}dt \\ \leq EV\left(\bar{x}(0)\right)+E\int_{0}^{t_{f}} \left(\bar{x}^{T}\bar{C}^{T}\bar{Q}\bar{C}\bar{x}+\left(\frac{\partial V(\bar{x})}{\partial\bar{x}}\right)^{T}\bar{f}_{w}(\bar{x},S,I_{M})\right. \\ \left.\text{ }+\frac{1}{2}\bar{f}_{w}(\bar{x},S,I_{M})^{T}\frac{\partial^{2}V(\bar{x})}{\partial\bar{x}^{2}}\bar{f}_{w}(\bar{x},S,I_{M})\right)dt \end{matrix}$ (B3)

Then, we get the following inequality

$\begin{matrix} E\int_{0}^{t_{f}} \bar{y}^{T}\bar{Q}\bar{y}dt \\ \leq EV\left(\bar{x}(0)\right)+E\int_{0}^{t_{f}} \left(\bar{x}^{T}\bar{C}^{T}\bar{Q}\bar{C}\bar{x}+\left(\frac{\partial V(\bar{x})}{\partial\bar{x}}\right)^{T}\bar{f}_{w}(\bar{x},S,I_{M})\right. \\ \left.\text{ }+\frac{1}{2}\bar{f}_{w}(\bar{x},S,I_{M})^{T}\frac{\partial^{2}V(\bar{x})}{\partial\bar{x}^{2}}\bar{f}_{w}(\bar{x},S,I_{M})\right)dt \end{matrix}$ (B4)

By the first inequality in (14), we get

$E\int_{0}^{t_{f}} \bar{y}^{T}\bar{Q}\bar{y}dt\leq EV\left(\bar{x}(0)\right)$ (B5)

By the fact

$EV\left(\bar{x}(0)\right)\leq\alpha$ (B6)

we can conclude

$J_{2}(S)\leq\alpha$ (B7)

That is, if the inequalities (14) and (B6) hold, then *J*_2_(*S*) is bounded by $\alpha$. Similarly, following (B1) and by Ito formula, we get

$\begin{matrix} dV(\bar{x})=\left(\frac{\partial V(\bar{x})}{\partial\bar{x}}\right)^{T}\left(\left(\bar{f}(\bar{x},S,I_{M})+\bar{H}\bar{v}\right)dt+\bar{f}_{w}(\bar{x},S,I_{M})d\bar{w}\right) \\ \text{ }+\frac{1}{2}\bar{f}_{w}(\bar{x},S,I_{M})^{T}\frac{\partial^{2}V(\bar{x})}{\partial\bar{x}^{2}}\bar{f}_{w}(\bar{x},S,I_{M})dt \end{matrix}$ (B8)

By the fact

$\left(\frac{\partial V(\bar{x})}{\partial\bar{x}}\right)^{T}\bar{H}\bar{v}\leq\frac{1}{4\beta}\left(\frac{\partial V(\bar{x})}{\partial\bar{x}}\right)^{T}\bar{H}\bar{H}^{T}\left(\frac{\partial V(\bar{x})}{\partial\bar{x}}\right)+\beta\bar{v}^{T}\bar{v}$ (B9)

Then, we get the following inequality

$\begin{matrix} E\int_{0}^{t_{f}} \bar{y}^{T}\bar{Q}\bar{y}dt \\ \leq EV\left(\bar{x}(0)\right)+E\int_{0}^{t_{f}} \left(\bar{x}^{T}\bar{C}^{T}\bar{Q}\bar{C}\bar{x}+\left(\frac{\partial V(\bar{x})}{\partial\bar{x}}\right)^{T}\bar{f}_{w}(\bar{x},S,I_{M})+\beta\right.\bar{v}^{T}\bar{v} \\ \left.\text{+}\frac{1}{4\beta}\left(\frac{\partial V(\bar{x})}{\partial\bar{x}}\right)^{T}\bar{H}\bar{H}^{T}\left(\frac{\partial V(\bar{x})}{\partial\bar{x}}\right)+\frac{1}{2}\bar{f}_{w}(\bar{x},S,I_{M})^{T}\frac{\partial^{2}V(\bar{x})}{\partial\bar{x}^{2}}\bar{f}_{w}(\bar{x},S,I_{M})\right)dt \end{matrix}$ (B10)

By the second inequality in (14) with *V*($\overline{x}$(*t*))>0 and *V*($\overline{x}$(0))=0, we get

$E\int_{0}^{t_{f}} \bar{y}^{T}\bar{Q}\bar{y}dt\leq E\beta\int_{0}^{t_{f}} \bar{v}^{T}\bar{v}dt$ (B11)

Then we obtain

$J_{\infty}(S)=\frac{E\int_{0}^{t_{f}} \bar{y}^{T}\bar{Q}\bar{y}dt}{E\int_{0}^{t_{f}} \bar{v}^{T}\bar{v}dt}\leq\beta$ (B12)

From (B7) and (B11), then the multi-objective problem in (10)-(12) becomes the multi-objective problem in (14).
