# Supplementary material for: Systematic Design of a Metal Ion Biosensor: A Multi-Objective Optimization Approach
Source: PLoS One. 2016 Nov 10;11(11):e0165911. doi: 10.1371/journal.pone.0165911 (PMC5104392; doi:10.1371/journal.pone.0165911)
Supplement: S3 File — (DOCX) [file pone.0165911.s003.docx]

**Supplementary Appendix C: Proof of Proposition 2**

First, we use the following fuzzy interpolation system

$\begin{matrix} d\bar{x}(t)=\sum_{p=1}^{L} \mu_{p}(z)\left(\left(\bar{A}_{p}\bar{x}(t)+\bar{H}\bar{v}(t)\right)dt+\bar{A}_{wp}\bar{x}(t)d\bar{w}(t)\right) \\ \bar{y}(t)=\bar{C}\bar{x}(t) \end{matrix}$ (C1)

in (16) to replace (7). Following the proof of Proposition 1 in Appendix B and by the fact that *EV*($\overline{x}$(*t_f_*))$\geq$0, we get the following result

$\begin{matrix} E\int_{0}^{t_{f}} \bar{y}^{T}\bar{Q}\bar{y}dt \\ \leq EV\left(\bar{x}(0)\right)+E\int_{0}^{t_{f}} \left(\bar{x}^{T}\bar{C}^{T}\bar{Q}\bar{C}\bar{x}+\sum_{p=1}^{L} \mu_{p}(z)\left(\left(\frac{\partial V(\bar{x})}{\partial\bar{x}}\right)^{T}\bar{A}_{p}\bar{x}(t)\right.\right. \\ \text{ }\left.\left.\text{ }+\frac{1}{2}\bar{x}(t)^{T}\bar{A}_{wp}^{T}\frac{\partial^{2}V(\bar{x})}{\partial\bar{x}^{2}}\bar{A}_{wp}\bar{x}(t)\right)dt\right) \end{matrix}$ (C2)

If we choose *V*($\overline{x}$)=$\overline{x}$*^T^P*$\overline{x}$, then

$\frac{\partial V(\bar{x})}{\partial\bar{x}}=2P\bar{x},\text{ }\frac{\partial^{2}V(\bar{x})}{\partial\bar{x}^{2}}=2P$ (C3)

then we get the following result from (C2)

$\begin{matrix} E\int_{0}^{t_{f}} \bar{y}^{T}\bar{Q}\bar{y}dt \\ \leq E\bar{x}(0)^{T}P\bar{x}(0)+E\sum_{p=1}^{L} \int_{0}^{t_{f}} \bar{x}^{T}\left(\bar{C}^{T}\bar{Q}\bar{C}+P\bar{A}_{p}+\bar{A}_{p}^{T}P\right. \\ \text{ }\left.\text{ }+\bar{A}_{wp}^{T}P\bar{A}_{wp}\right)\bar{x}dt+E\int_{0}^{t_{f}} \bar{v}^{T}\bar{v}dt \end{matrix}$ (C4)

If

$\bar{C}^{T}\bar{Q}\bar{C}+P\bar{A}_{p}+\bar{A}_{p}^{T}P+\bar{A}_{wp}^{T}P\bar{A}_{wp}<0$ (C5)

then we get

$E\int_{0}^{t_{f}} \bar{y}^{T}\bar{Q}\bar{y}dt\leq E\bar{x}(0)^{T}P\bar{x}(0)$ (C6)

By the fact

$E\bar{x}(0)^{T}P\bar{x}(0)\leq\alpha$ (C7)

we can conclude

$J_{2}(S)\leq\alpha$ (C8)

That is, if the inequalities (C6) and (C8) hold, then *J*_2_(*S*) is bounded by $\alpha$. Similarly, by the following inequality

$\begin{matrix} E\int_{0}^{t_{f}} \bar{y}^{T}\bar{Q}\bar{y}dt \\ \leq EV\left(\bar{x}(0)\right)+E\int_{0}^{t_{f}} \left(\bar{x}^{T}\bar{C}^{T}\bar{Q}\bar{C}\bar{x}+\sum_{p=1}^{L} \mu_{p}(z)\left(\left(\frac{\partial V(\bar{x})}{\partial\bar{x}}\right)^{T}\bar{A}_{p}\bar{x}(t)\right.\right. \\ \text{ }\left.\left.\text{ +}\left(\frac{\partial V(\bar{x})}{\partial\bar{x}}\right)^{T}\bar{H}\bar{v}+\frac{1}{2}\bar{x}(t)^{T}\bar{A}_{wp}^{T}\frac{\partial^{2}V(\bar{x})}{\partial\bar{x}^{2}}\bar{A}_{wp}\bar{x}(t)\right)dt\right) \end{matrix}$ (C9)

and by the fact

$\left(\frac{\partial V(\bar{x})}{\partial\bar{x}}\right)^{T}\bar{H}\bar{v}\leq\frac{1}{4\beta}\left(\frac{\partial V(\bar{x})}{\partial\bar{x}}\right)^{T}\bar{H}\bar{H}^{T}\left(\frac{\partial V(\bar{x})}{\partial\bar{x}}\right)+\beta\bar{v}^{T}\bar{v}$ (C10)

we get the following inequality

$\begin{matrix} E\int_{0}^{t_{f}} \bar{y}^{T}\bar{Q}\bar{y}dt \\ \leq E\bar{x}(0)^{T}P\bar{x}(0)+E\sum_{p=1}^{L} \int_{0}^{t_{f}} \bar{x}^{T}\left(\bar{C}^{T}\bar{Q}\bar{C}+P\bar{A}_{p}+\bar{A}_{p}^{T}P\right. \\ \text{ }\left.\text{ +}\frac{1}{\beta}P\bar{H}\bar{H}^{T}P+\bar{A}_{wp}^{T}P\bar{A}_{wp}\right)\bar{x}dt+E\beta\int_{0}^{t_{f}} \bar{v}^{T}\bar{v}dt \end{matrix}$ (C11)

If

$\bar{C}^{T}\bar{Q}\bar{C}+P\bar{A}_{p}+\bar{A}_{p}^{T}P\text{+}\frac{1}{\beta}P\bar{H}\bar{H}^{T}P+\bar{A}_{wp}^{T}P\bar{A}_{wp}<0$ (C12)

then we get

$E\int_{0}^{t_{f}} \bar{y}^{T}\bar{Q}\bar{y}dt\leq E\bar{x}(0)^{T}P\bar{x}(0)+E\int_{0}^{t_{f}} \bar{v}^{T}\bar{v}dt$ (C13)

If $\overline{x}$(0)=0, we get

$E\int_{0}^{t_{f}} \bar{y}^{T}\bar{Q}\bar{y}dt\leq E\beta\int_{0}^{t_{f}} \bar{v}^{T}\bar{v}dt$ (C14)

Then we obtain

$J_{\infty}(S)=\frac{E\int_{0}^{t_{f}} \bar{y}^{T}\bar{Q}\bar{y}dt}{E\int_{0}^{t_{f}} \bar{v}^{T}\bar{v}dt}\leq\beta$ (C15)

By Schur complement, the inequalities in (C7), (C5) and (C12) are equivalent to the LMIs in (18), (19), and (20), respectively, i.e. if the LMIs in (18), (19), and (20) have a common solution *P*>0, then the output of an engineered multi-objective H_2_/H_∞_ QS-based metal ion biosensor will optimally match the specified reference output and optimally filter parameter fluctuations and cellular noise.
